# Geographical Variations in the Interaction of Relative Age Effects in Youth and Adult Elite Soccer

**DOI:** 10.3389/fpsyg.2017.00278

**Published:** 2017-03-07

**Authors:** Christina Steingröver, Nick Wattie, Joseph Baker, Werner F. Helsen, Jörg Schorer

**Affiliations:** ^1^Department of Sport and Motion, Institute of Sport Science, University of OldenburgOldenburg, Germany; ^2^Faculty of Health Sciences, University of Ontario Institute of Technology, OshawaON, Canada; ^3^School of Kinesiology and Health Science, York University, TorontoON, Canada; ^4^Department of Kinesiology, Research Centre for Movement Control and Neuroplasticity, KU LeuvenLeuven, Belgium

**Keywords:** relative age effects, constituent year effect, professional soccer, talent development, expertise development, age grouping policies

## Abstract

Selection biases based on the use of cut-off dates and the timing of athletes’ birthdates have been termed relative age effects. These effects have been shown to differentially affect individuals involved in sport. For example, young male soccer players born early in their age group are overrepresented in elite teams while studies in adult soccer indicated potential carry-over effects from talent development systems. This two-study approach focuses on the processes within multi-year age groups in youth and adult elite soccer and on the role of players’ age position within the age band with regard to players’ birth year and birth month. Study 1 tests for an interaction of two different types of relative age effects among data from participants in the last five Under-17 FIFA World Cups (2007–2015). Analyses revealed a significant global within-year effect and varying birthdate distributions were found between confederations. Even stronger effects were found for constituent year effects. For the total sample, a multi-way frequency analysis (MFA) revealed an interaction with a pattern of a stronger within-year effect for the younger year group. This study highlights the need to consider interactions between different types of age effects. The main aim of Study 2 was to test for carry-over effects from previously found constituent year effects among players participating in the 2014 soccer World Cup and, therefore, to test for long-term effects of age grouping structures used during earlier stages of talent development. A secondary purpose of this study was to replicate findings on the existence of within-year effects and to test whether effects vary between continental confederations. No significant interaction between constituent year and within-year effects was shown by the MFA among the World Cup sample and previous findings on varying within-year effects were replicated. Results indicate that long-term effects of age grouping structures in earlier high-level talent development structures exist.

## Introduction

For many sport administrators, the problem of how to group children for equal and safe competition in sport is usually solved by creating age groups, typically through the use of selection dates (e.g., January 1st). In this approach, a player born in January is almost 12 months older than their prospective teammate born in December. Research has demonstrated that systems using selection dates create learning environments in which children’s age relative to the selection date influences his or her chance of having success in sport, where significant overrepresentations of players born in the first quartile have been identified repeatedly (Barnsley et al., 1985; Barnsley and Thompson, 1988; Cobley et al., 2009; Baker et al., 2010; Wattie et al., 2015). Due to greater chronological age, relatively older athletes have an increased likelihood of advanced maturation and advanced physical characteristics (Malina et al., 2007). The *maturation-selection hypothesis* proposes these characteristics enhance performance (e.g., in soccer) so that relatively older individuals show an increased likelihood of both a performance and selection advantage (Schorer et al., 2013). As such, relatively older players have a higher chance of accessing higher levels of competition and increased training conditions (Helsen et al., 1998b).

During the last three decades the body of literature on relative age effects in soccer has continuously grown. As a result, soccer is among the most studied sports in relative age research (Helsen et al., 1998b, 2012; Musch and Hay, 1999; Vaeyens et al., 2005; Malina et al., 2007; Cobley et al., 2008; Nakata and Sakamoto, 2011). Cobley et al. (2009) reported that about 30% of the studies investigating relative age effects have focused on soccer. While considerable research has documented the existence of RAEs in soccer (and many other sports; see Cobley et al., 2009), Wattie et al. (2015) suggested that in order to have a better theoretical and applied understanding of RAEs, it is necessary to also consider how different constraints (individual, task, and environmental) influence the expression of specific RAEs. One important environmental/task constraint affecting RAEs may be the different *types* of age grouping policies used in sport systems, such as the 2-year systems in German handball (Schorer et al., 2013), soccer (Lames et al., 2008), and Canadian junior ice hockey (Wattie et al., 2010) and the 3-year bands in German youth basketball (Steingröver et al., 2016). However, surprisingly little is known about how the effects of different age grouping systems interact and how they influence players’ attainment opportunities in systems where policies determine the use of 2- or more-year age bands.

A recent categorization of relative age effects by Schorer et al. (2013) showed interacting relative age effects in handball, namely *within-year effects* and *constituent year effects*; a mechanism that may also be relevant for understanding age effects in soccer. *Within-*1*-year* and *within-*2*-year effects* characterize typical relative age effects showing an overrepresentation of relatively older players within selection cohorts spanning a 12 and 24 months range, respectively. Alternatively, *constituent year effects* can also exist in multi-year age bands. In these systems, athletes can have within-year effects (i.e., typical relative age effects) and an athlete’s age relative to the other members of the age cohort changes every subsequent season as they move through that multi-year age band. Examples of such a dynamic youth sport system are the 2-year bands in Canadian youth ice hockey (Wattie et al., 2010) and the two 3-year bands in elite German youth basketball (Steingröver et al., 2016). Youth sport systems using this kind of age grouping often show overrepresentations of the oldest age cohort. At the adult level, the opposite trend is shown in the 5-year bands in masters’ sport (Medic et al., 2007) where overrepresentations of the youngest cohorts are demonstrated.

While within-year effects and constituent year effects have predominantly been considered separately (mainly the within-year effect), a recent study by Steingröver et al. (2016) showed that these effects interacted in basketball. The interaction between constituent year and within-1-year effects in the Under-16 3-year age band showed reduced within-1-year effects with increasing absolute age. Results indicated that development policies influence young players’ development of sport expertise in basketball, especially the development of relatively younger players who often enter the talent development system 1 or 2 years later than their relatively older peers. However, once players enter the system in the Under-16 league, relatively younger players seem at a lower risk of dropping out of the system. This study provided new insight regarding how the regulations of a talent development system constrain young athletes’ entrance opportunities into the system and their likelihood of continuing onto the highest competition levels.

Our main aim in the current study was to investigate the potential interactions among different forms of relative age effects in elite male soccer in two studies. In Study 1, we focus on youth soccer, more specifically on the Under-17 World Cups between 2007 and 2015. The study investigates whether players’ participation opportunities are affected by their within-year relative age (birth month) and their constituent year timing (birth year). In Study 2, we investigate the maintenance of these interactions in adult soccer at the 2014 FIFA (i.e., Fédération Internationale de Football Association) World Cup in Brazil and examine potential long-term effects of age grouping policies and tournament timing in elite youth soccer.

## Study 1: Geographical Variations of the Interactions of Constituent Year and Within-Year Effects Among Junior World Cup Soccer Players

Within-year effects have been found among different age groups in various countries and soccer contexts. For youth club level soccer players, relative age effects have been shown in France (Delorme et al., 2010), Germany (Augste and Lames, 2011) and Belgium (Helsen et al., 2000). The same pattern was seen among junior national teams (Penna et al., 2012; Sallaoui et al., 2014). Originally, Barnsley et al. (1992) revealed that 46% of players participating in the Under-17 World Cup in 1989 were born in the first quartile and only 8% in the last quartile of the selection year. In Europe, Helsen et al. (2005) showed overrepresentations of players born in the first quarter of the selection year (from January to March) for all national youth selection teams at the Under-15, Under-16, Under-17, and Under-18 age categories in 10 European countries. In the United States, Vincent and Glamser (2006) tested for overrepresentations of relatively older players among the Olympic Development Program and found a strong relative age effect in male state, regional and national team players. More recently, Williams (2010) demonstrated an overall within-year effect and varying birthdate distributions among the multi-nation sample participating in the six subsequent Under-17 World Cups between 1997 and 2007. However, this study took a closer look at the sample composition and revealed that not only age-appropriate players participated in the World Cups investigated but that the samples emerged as multi-age groups including *underage* players (Williams, 2010). As such, this study was among the first to indirectly investigate constituent year effects in soccer, but did not test for the interaction of those two effects.

Surprisingly, comparatively little research has focused on countries outside of Europe and North America (Cobley et al., 2009) and to the best of our knowledge few studies have considered relative age effects in the same sport around the globe. Among the few existing studies, Williams (2010) investigated the birthdate distribution of players participating in six subsequent FIFA Under-17 World Cups. The results revealed a clear overrepresentation of relatively older players for every tournament and a chronologically increasing percentage of participants born in the early months. This trend is interesting since FIFA proposed a change of cut-off dates in 1997 and defined January 1st as the consistent cut-off date for international competitions (for effects resulting from this shift in within-year effects see Helsen et al., 2000; Cobley et al., 2008). As such, many players developed in systems using different cut-off dates such as August 1st. Nevertheless, findings from Williams (2010) suggest an overrepresentation of January to March born players. Differentiating by continental confederations, five out of six confederations demonstrated skewed birth date distributions showing an overrepresentation of players born early in the competition year. Only for the African confederation an inverse distribution was revealed (Williams, 2010). To be more precise, Williams (2010) demonstrated an overrepresentation of quartile 4 born players for 14 out of 19 African national teams qualified for the six investigated World Cups between 1997 and 2007. He concluded this phenomenon was unique to West African nations but also requested further research to test whether inverse effects were specific to a certain region or consistent across the continent. The current study tries to extend this research through a different geographical approach.

The first aim of this study was to close the research gap with regard to the existence of both within-year and constituent year effects in worldwide soccer by examining data from the last five Under-17 World Cups in male soccer and to replicate findings from Williams (2010). Due to the large amount of time that has passed since FIFA demanded to unify the cut-off dates in 1997, players should no longer be affected by diverging cut-off dates that were formerly used in development systems (e.g., German soccer used August 1st before 1997). As such, it is interesting to ask whether players’ birthdates are even more skewed with increasing time to the determination of January 1st as a cut-off date.

The main aim of this study was to examine the possible interaction of within-1-year effects and players’ constituent year. While within-year and constituent year effects have been investigated separately in soccer, the interaction of both effects has never been tested in this sport. Given that the pattern of within-1-year effects was shown to differ between confederations in the study by Williams (2010), differentiating analyses need to be conducted to get a clearer picture. We hypothesized that within-year effects would exist and that older year groups would be overrepresented in comparison to younger ones. Due to the fact that the change of cut-off dates did not affect players in the current sample, effect sizes were assumed to increase over time. Considering the different 1 year age cohorts in the sample and with regard to the interaction of players’ constituent year and within-1-year effects, we expected that within-1-year effects would get smaller with increasing age (i.e., with increasing constituent year).

### Materials and Methods Study 1

#### Sample

For this study, players’ birthdate and nationality were obtained via official FIFA websites^1^ for the male Under-17 soccer world championships from 2007 to 2015. In contrast to the soccer world championships at the adult level, the Under-17 soccer world championships take place every 2 years and, as a result, our sample includes all players from five subsequent tournaments. Among the Under-17 age group, FIFA operates with the continental confederations: AFC (Asia), CAF (Africa), CONCACAF (North-central America), CONMEBOL (South America), OFC (Oceania), and UEFA (Europe). Each of the 24 national teams participating in the World Cup is asked to nominate 21 players resulting in a total sample of 2512 players. Within this sample, the representation of confederation varies greatly (**Table 1**). This variation can be explained by FIFA’s organizational procedure, which invites one team from Oceania, four teams from Africa, Asia, North-central America, and South America and six teams from Europe as well as the respective host country.

**Table 1:** Number of participating national teams per confederation among the World Cups between 2007 and 2015.

	AFC	CAF	CONCACAF	CONMEBOL	OFC	UEFA
2007 Korea	5	4	5	4	1	5
2009 Nigeria	4	5	4	4	1	6
2011 Mexico	4	4	5	4	1	6
2013 UAE	5	4	4	4	1	6
2015 Chile	4	4	4	5	1	6
Total number of teams	22	21	22	21	5	29
Total number of players	462	438	461	441	105	605
CY 3 and 4 excluded	456	403	458	441	105	605

#### Variables

FIFA has positioned January 1st as the cut-off date in junior tournament regulations^2^. For within-year effects (i.e., typical relative age effects) birth dates were recoded into four quartiles (Q1: January – March, Q2: April – June, Q3: July – September, Q4: October – December). Additionally, to check for constituent year effects, each player’s birth year was recoded to reflect his birth year position into year 1 (for the underage players) or year 2 (for the older players being in the regular Under-17 year). A few nations also included players who were 2 or 3 years under age. As such, theoretically four constituent year categories would have to be included into the analysis. Given that the number of all players more than 1 year under age was less than 1.8% of the sample, we excluded them from further analysis.

#### Analyses

Against the backdrop of previous studies revealing inverse within-year effects for the African confederation and the notion that these findings might be unique for West African national teams (Williams, 2010) separate analyses were conducted for the Northern nations of the CAF and for those of central and southern Africa, hereafter referred to as sub-Saharan nations, to gain further insight into possible mechanisms driving these effects. This division into two sub-samples is due to peoples’ assignment to *culture continents* (Kolb, 1962). The division of culture continents differs from the common continents and provides an alternative definition of regions that significantly differ from their neighboring regions with regard to political and economic structures. This approach (Kolb, 1962) subdivides the African continent into two large cross-border regions: *sub-Saharan Africa* and the *Orient* including the North African regions. Although this division is not without controversy (Popp, 2003), it potentially enables a more detailed insight into the environmental constraints (Wattie et al., 2015) that might influence the emergence of relative age effects in soccer. This approach focuses on broad sociocultural characteristics such as economics, political systems, and religion that influence peoples’ resulting way of life (Kolb, 1962).

IBM SPSS Statistics 23.0 and G^∗^Power 3.1 were used for statistical analyses (Faul et al., 2007). Asymptotic chi-square analyses were used to consider different distributions among relative age quartiles and to test for differences among the number of participants in their first or second constituent year, respectively. To test for an interaction of both effects, a multi-way frequency analysis (MFA) was conducted (Tabachnick and Fidell, 2013; for an application see also Steingröver et al., 2016). Due to the multi-nation sample underlying the current investigation, it was not possible to take into consideration potential differences in birth rates per month that might exist between countries. Therefore, an equal distribution of births across all months and years was assumed for all analyses.

### Results Study 1

#### Global Results

**FIGURE 1:**
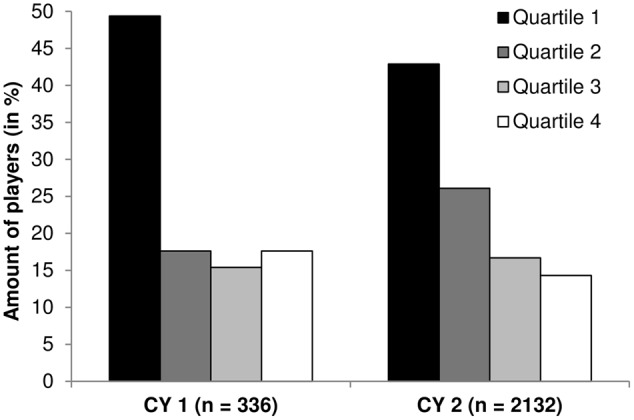
**Birthdate distribution and participation rates of U-17 soccer players.** CY, Constituent year; Separate chi-square analyses for the younger constituent year (CY 1) and the older constituent year (CY 2) revealed significant within-one-year effects for each year and showed a decrease of effect sizes by 0.11 from year 1 to year 2.

For the total sample of players participating among Under-17 World Cups between 2007 and 2015, stepwise backward elimination selection produced a model that included the interaction of constituent year effects and within-1-year effects, χ^2^ (3, 2468) = 14.43, *p* = 0.002. To test for skewed birthdate distributions, asymptotic chi-square analyses were calculated. For the overall sample, a significantly skewed birthdate distribution favoring relatively older players was revealed, χ^2^ (3, *n* = 2468) = 522.12, *p* < 0.001, *w* = 0.45. Separate chi-square analyses for the younger constituent year (year 1 = CY 1), χ^2^ (3, *n* = 336) = 107.12, *p* < 0.001, *w* = 0.56, and older constituent year (year 2 = CY 2), χ^2^ (3, *n* = 2132) = 429.93, *p* < 0.001, *w* = 0.45, revealed significant within-1-year effects for each year and showed a decrease of effect sizes by 0.11 from years 1 to 2 (see **Figure 1**).

Even stronger effects were found in the constituent year effect analyses among the whole sample of players, χ^2^ (1, *n* = 2468) = 1306.98, *p* < 0.001, *w* = 0.70, testing for an overrepresentation of players in a certain 1-year age band (i.e., constituent year). The sample sizes of the two age cohorts emphasized the strong overrepresentation of the oldest age band: 86.4% of players were born in the older year.

#### Differentiating between Confederations

In a second step, analyses were differentiated between confederations. Testing for an interaction of different relative age effects among the overall sample of each confederation as well as for the two African cultural continents, a significant interaction of constituent year effects and within-1-year effects was only shown for the Asian confederation, χ^2^ (3, *n* = 456) = 10.39, *p* < 0.05, where analyses revealed a significant overrepresentation of quartile 1, χ^2^ (3, *n* = 456) = 94.07; *p* < 0.001, *w* = 0.44, and of the older constituent year, χ^2^ (3, *n* = 456) = 213.47, *p* < 0.001, *w* = 0.66. Separate chi-square analyses for the younger (constituent year 1), χ^2^ (3, *n* = 72) = 46.44, *p* < 0.001, *w* = 0.80, and older age bands (constituent year 2), χ^2^ (3, *n* = 384) = 61.50, *p* < 0.001, *w* = 0.39, revealed significant within-1-year effects for each year and showed a decrease in effect size by 0.41 from years 1 to 2.

In a further step, results were differentiated between confederations and geographic regions to analyze the overall effect in more detail. Analyses revealed the expected within-year effects in five out of six confederations, χ^2^ (3, *n* = 105–605) = 22.96–221.57, *p* < 0.01, *w* = 0.44–0.60, with the exception of the African confederation. Differentiating between the North African nations and the sub-Saharan nations, results showed a different picture. A strong typical within-year effect was shown for the North African nations, χ^2^ (3, *n* = 83) = 56.13, *p* < 0.01, *w* = 0.82. In the sub-Sahara region a significant inversed within-year effect was found, χ^2^ (3, *n* = 441) = 38.18, *p* < 0.01, *w* = 0.33. Differentiating between the continental confederations, all regions revealed an overrepresentation of the older age cohort (i.e., constituent year 2) within the investigated 2-years age band, χ^2^ (1, *n* = 105–609) = 28.80–512.81, *p* < 0.01, *w* = 0.31–0.91.

Last, analyses were conducted separately for each of the five tournaments (see **Table 2**). No interactions were revealed by the MFA. The main aim of this differentiation was to test whether a potential within-year effect decreased or increased over the years and therefore to shed light on the role of players’ age position within the age band. Results revealed significant results for each of the five competitions and showed medium to large effect sizes, χ^2^ (3, *n* = 488–502) = 67.67–146.21, *p* < 0.001, *w* = 0.37–0.54. As can be seen in **Table 2**, the effect sizes increased, albeit not consistently, from a medium to a large effect over the years. Furthermore, constituent year effects showing an overrepresentation of the older age cohort within multi-year age groups were revealed for every World Cup between 2007 and 2015, χ^2^ (1, *n* = 488–502) = 231.34–285.24, *p* < 0.001, *w* = 0.69–0.76.

**Table 2:** Within-year effects separated by Under-17 World Cup.

World Cup	Overall within-year effect	Effect size *w*
Korea 2007	χ^2^ (3, *n* = 494) = 75.67, *p* < 0.01	*w* = 0.39
Nigeria 2009	χ^2^ (3, *n* = 488) = 112.90, *p* < 0.01	*w* = 0.48
Mexico 2011	χ^2^ (3, *n* = 491) = 67.67, *p* < 0.01	*w* = 0.37
UAE 2013	χ^2^ (3, *n* = 493) = 146.21, *p* < 0.01	*w* = 0.54
Chile 2015	χ^2^ (3, *n* = 502) = 133.71, *p* < 0.01	*w* = 0.51

### Discussion Study 1

Previous research in soccer suggests an individual’s relative age within the respective age groups influences the likelihood of both participation and success (Helsen et al., 2005). One aim of this study was to examine data from the last five Under-17 World Cups in male soccer (2007–2015) and to replicate findings on within-year effects on the highest level of junior soccer competition (Williams, 2010; Sallaoui et al., 2014). The current findings support this notion with a significant overall within-year effect favoring the relatively older players.

Another aim of our study was to consider the existence of constituent year effects among Under-17 World Cup participants of the last 10 years. Constituent year effect analyses revealed an overall overrepresentation of the older age cohort within multi-year age groups: the sample sizes of the age cohorts (*appropriate age* and *younger*) within this multi-year age band highlight the strong overrepresentation of the oldest age band (84.87%) compared to the younger age bands (15.13%).

Differentiating between the continental confederations, all six confederations revealed a significant within-year effect. However, the importance of considering geographical variations among within-year effects in youth soccer was demonstrated for the African confederation. While a significant within-year effect was shown for the entire CAF sample, analysis revealed stronger significant within-year effects with differing directions as soon as the sample was subdivided into cultural continents (Kolb, 1962). Separate analyses for North and sub-Saharan Africa revealed a strong typical within-year effect for North African participants while an inverse effect was replicated (Williams, 2010; Sallaoui et al., 2014) for the sub-Saharan national teams. These outcomes reflect the current state of research on relative age effects and replicate previous findings on within-year effects in soccer (Cobley et al., 2008; Helsen et al., 2012). Although results cannot be traced back definitively to the last determination of January 1st as a cut-off date in 1997, it is interesting that results appear to suggest that during the last five tournaments (or during the last 10 years) the effect sizes of the overall within-year effects increased from 0.39 to 0.51. This finding might be due to the fact that previously used selection dates have increasingly lost their influence on players in the current sample since the cut-off date change mandated by FIFA. The significant within-year effects support the notion that relative age is still an important constraint on early success and high-level representation among soccer players over the world.

With regard to constituent year effects differentiated by confederation, an overrepresentation of the older age cohort within the 2-year age band was demonstrated for every confederation and across the cultural continents among CAF members. This is in line with results from the small amount of previous studies in other sports using similar age grouping structures (Lames et al., 2008; Wattie et al., 2010; Schorer et al., 2013; Steingröver et al., 2016). Although geographical variations were shown among birthdate distributions, all confederations were affected by both types of relative age effects. FIFA regulations permit national teams to compete as two- or more multi-year age bands. However, the underlying system structure fosters a high participation rate of those players born in the appropriate birth year for an Under-17 championship and therefore a constituent year effect in Under-17 tournaments. Results are in line with findings from van den Honert (2012) who demonstrated among Australian soccer players that players’ age (i.e., players’ birth year in a multi-year age band) influences their chances to participate in a Under-17 and Under-20 World Cup. However, the consequences of age grouping structures and of the tournaments’ timing for youth players’ future development require further investigation (van den Honert, 2012). The importance of players’ constituent year becomes clear as soon as a closer look is taken at the tournament’s 2-year rhythm: the FIFA Under-17 World Cup takes place every 2 years. Although the junior national teams are designed as flexible age groups in which each player can be in the younger cohort within a 2-year age band in one playing season but the older in the subsequent season, this does not make a difference for the majority of soccer talents. Players participating in the Under-17 world championship as a member of the age cohort that is 1 year younger than the appropriate age group within the multi-year age band will not have the chance to benefit from potential advantages of being among the relatively oldest participants in a tournament; in the respective year that they become the oldest age cohort no Under-17 World Cup takes place. Their next chance to compete in a FIFA world championship will be provided among a Under-20 championship 2 years later – then as a relatively younger player once again, namely as an Under-18 player. Another 2 years later players born in an odd year have the chance to participate in a World Cup as a member of the oldest age group (van den Honert, 2012). Originally designed as a system where players have a varying constituent year and a within-year relative age, the tournament timing determines players’ unique position within the multi-year squad. Consequently, the benefit of moving to an older age cohort in the season following the World Cup is limited. In future work, it would be interesting to investigate whether players making it to the Under-17 World Cup at an early point in time benefit from outstanding playing skills or early maturation or both.

The main aim of this study was to find out whether an interaction between within-1-year effects and constituent year effects exists in international elite youth soccer. Although results only reached significance for the interaction among the overall sample and for the Asian confederation, results highlight the need to consider interactions between different types of relative age effects in multi-year age bands. To our knowledge, the interaction of constituent year effects and within-year effects has not been investigated previously in soccer and results highlight the complexity of age effects in sport and its underlying systems. The pattern shows a slightly stronger within-year effect for the younger 1-year age groups (overall: *w*_cy1_ = 0.56 > *w*_cy2_ = 0.45; Asia: *w*_cy1_ = 0.80 > *w*_cy2_ = 0.39) and therefore confirms our hypothesis that within-1-year effects get smaller with increasing age. It seems that a player’s constituent year within national teams does not only influence their participation opportunities but also the role of their relative age within the 1-year age band. The study by Steingröver et al. (2016) demonstrated an interaction between constituent and within-1-year effects in elite German youth basketball, which supports the notion that with increasing absolute age, relatively younger basketball players seem more able to overcome their age-related disadvantages. Or to put it differently, only the most advanced of the younger constituent year possess a chance of being selected at early stages of talent development. At later stages, the relatively oldest from the younger constituent year may no longer stand out against their peers as obviously as at earlier stages of talent development so relatively younger players’ chances of being selected increase. Although effect sizes decrease with increasing age among the samples where interactions were shown, the medium effect sizes are still quite common in relative age research (Cobley et al., 2009). As such, relative age advantages apparently remain a strong influence on players’ opportunities to reach the highest level of junior soccer competition. Overall, chronological age seems to be more important than the relative age within a birth year, but the comparatively high effect sizes for within-year effects indicate that players are still far from overcoming age-related disadvantages.

This study shows nicely that within-year effects showing an overrepresentation of quartile 1 born players might not be as global as previously thought. The specific pattern differences in sub-Saharan Africa and North Africa provide further insight into the nuances of relative age effects, although further analyses are required to understand the inverse trends among sub-Saharan CAF nations. The current investigation of international samples highlighted the benefit of interdisciplinary approaches and the importance of subdivisions within complex samples to reveal as much as possible of a certain relative age effect’s spectrum. A limitation of an overall testing for within-year effects analyzing birthdates across all confederations is that it would have covered the inverse results in the CAF. Additionally, even a confederation-wise analysis would have produced results that reflect a misleading picture of the investigated within-year effect in the total sample of participation CAF nations: due to the counteracting, balancing effect of the birthdate distribution among players from sub-Saharan Africa on the total sample, the overall within-year effect would have been less pronounced.

Furthermore, results indicate the importance of system-specific backgrounds and the need to consider specific environmental constraints (Wattie et al., 2015). In talent development systems such as the FIFA Under-17 structure, where players compete in dynamic multi-year age bands, data analyses of players’ birth month might not be precise enough to capture the complexity of relative age effects in sport systems. Such an analysis is very specific and captures only a short time span of national squad competition in international soccer. It will also be important to consider any long-term impacts of interactions between constituent year and within-year effects as well as players’ constituent year timing on their future athlete development and success at the highest level of competition. The age cohorts investigated by Williams (2010) included players who should be at their peak performance now which is generally reached in soccer at the age of 27–30 years (Boone et al., 2012). Therefore, in case players stayed in the system, varying birthdate distributions should still be detectable in the recent World Cup sample.

While this study highlights areas for further work on age-related effects in elite junior soccer, the analyses were not without limitations. First, a MFA requires cell sizes of ≥5 (Tabachnick and Fidell, 2013), and it must be noted that some cell sizes, especially representing the younger players in their first constituent year, did not reach this threshold of players born in the respective quartiles in differentiating analyses. Therefore, a condition for the application of a MFA was not realized in some cases, which might cause problems within the data analyses; with regard to the small cell sizes among players in constituent year 1, the program based adjustment of cell sizes might balance the distribution too strongly and therefore affect MFA results. The most significant limitation of this study, however, was our very limited knowledge about the talent development systems in some CAF nations. Due to the decisive role that players’ birthdates necessarily play in relative age research, the statistical significance heavily relies on the accuracy of the reported birthdates. Possible mechanisms that might lead to errors in reporting an individual’s birth date are manifold (Ndong et al., 1994) and will be discussed in the general discussion at the end of this paper. Future investigations may also consider the influence of additional environmental constraints (Wattie et al., 2015) on the current findings on relative age effects in African soccer, such as regional and national talent identification and development systems and the philosophy within the system. Relatively younger players are thought to benefit in the long-term from being initially younger (Wattie et al., 2007; Gibbs et al., 2012) and from being possibly initially disadvantaged (McCarthy and Collins, 2014). Even though the reasons relatively younger players have more positive outcomes later in their sporting career are not known (Schorer et al., 2009), research suggests younger players may benefit from the technical and psychological challenge of competing with more mature teammates (Ashworth and Heyndels, 2007; Hirose, 2009). As such, research might focus on whether the African soccer systems foster opportunities for relatively younger players to start playing soccer at an early age and, importantly, keep players involved in soccer after puberty ends. Not least because of the fact that CAF members provided three of the last five Under-17 world champions and five of 10 final teams within this period, future research on the impact of relative age on talent development and achievement opportunities should make an increased effort to investigate the constraints influencing the birthdate patterns shown for large parts of the African confederation.

## Study 2: Tracing Relative Age Effects in Elite Soccer: Analysis of Geographical Variations of Relative Age Effects Among FIFA 2014 World Cup Players

In adult soccer, several studies have shown within-year effects with an overrepresentation of relatively older players, indicating long-term carry-over effects from the age grouping policies in talent development and youth sport systems (Barnsley et al., 1992; Richardson and Stratton, 1999; Edgar and O’Donghue, 2003). Results from Williams (2010) and Study 1 indicate that the (inter-)national Under-17 talent development system favors players born in the early months of the selection year and members of the older 1-year age group. Given these results, the purpose of this study was to test in a quasi-longitudinal study the long-term impact of within-year effects and of players’ constituent year timing.

A large number of studies have examined within-year effects separately. To the best of our knowledge the potential long-term effects of players’ constituent year timing in talent development systems in soccer have not been investigated. Therefore, one aim of Study 2 is to test whether players born (a) in the later months of the year (i.e., quartile 4) and (b) in the former younger 1-year age cohorts are still underrepresented in the 2014 World Cup. Based on the continuously skewed birthdate distribution demonstrated for Under-17 World Cups in Study 1 and by Williams (2010) over the last 20 years, there is a real possibility of long-term outcomes resulting in carry-over effects among recent adult tournaments. As such it is necessary to determine whether patterns and effects from the national talent development path carry-over to the adult world elite soccer. The current state of research provides evidence for the carry-over of within-year effects into elite adult soccer, thereby emphasizing the persistence of this finding. Studies of potential long-term consequences of constituent year effects and the interaction of different types of relative age effects demonstrated in Study 1 contribute to our understanding of the carry-over of different relative age effects detected in talent development systems.

Differentiating between continental confederations in Study 1, within-year effects were shown to differ considerably. At the same time, all regions revealed an overrepresentation of the older 1-year age cohort (see also Williams, 2010). Therefore, another aim of this study was to replicate findings on geographically varying within-year effects. Within-year effects differing between continental confederations might also be based on system differences such as strategies with regards to talent selection and talent development structures. It is possible that these strategies may prevent the emergence of relative age effects, and therefore they definitively seem worthy of exploration.

Based on the findings on within-year effects in elite adult soccer (Barnsley et al., 1992; Edgar and O’Donghue, 2003) we hypothesized an overall overrepresentation of quartile 1 born players indicating a typical within-year effect among players participating in the FIFA World Cup 2014 in Brazil. In line with previous research in soccer (and other sports), Study 1 suggests older year groups are overrepresented in comparison to younger ones in the Under-17 World Cup sample indicating a constituent year effect (Lames et al., 2008; Schorer et al., 2013; Steingröver et al., 2016). However, the possible carry-over of these effects has not yet been investigated in adult soccer; therefore, we explore this in the current study.

However, on the basis of Study 1, trends and effect sizes should vary across the continental confederations. The imbalance in the number of studies conducted for each confederation, showing a strong emphasis on Europe and North America (Cobley et al., 2009), makes it difficult to predict the outcomes of such a geographical variation but typical relative age distributions are expected for all confederations except sub-Saharan Africa where literature and Study 1 have suggested the presence of an inverse birthdate distribution (Williams, 2010; Sallaoui et al., 2014).

### Materials and Methods Study 2

The participants were 736 male soccer players who were nominated for the World Cup 2014 in Brazil. Each of the 32 soccer associations that qualified for the final competition provided a final list of 23 players 2 weeks before the tournament started. To examine within-year effects in this sample, players’ birth dates were collected on the basis of the 2014 FIFA World Cup Brazil^TM^ list of players, which is available on the tournament’s official website^3^. Subsequently, birth dates were categorized into birth quartiles according to the January 1st cut-off date commonly used in national junior and talent development systems. Therefore, quartile 1 includes players born in January – March, quartile 2 in April – June, quartile 3 in July – September, and quartile 4 includes players born in October – December. Just a few national soccer organizations such as the German DFB or the English FA conducted a change of age group categorization in the past or use different cut-off dates currently. In Germany, August 1st was applied to distribute players into age groups prior to 1996 and then rearranged to January 1st. Consequently, an individual’s classification to a certain birth quartile shifts and was adjusted accordingly in the data collection (Quartile 1: August – October, Quartile 2: November – January, Quartile 3: February – April, and Quartile 4: May – July). As a result, for consistency of recording, Quartile 1 is at the beginning, while Quartile 4 is at the end of the selection period. Using the example of Germany, this adjustment was conducted for players born after 1981 (≤15 years of age in 1996), because they were affected by an altered structure within the youth soccer system they were part of (Cobley et al., 2008).

To check for a potential carry-over effect regarding an underrepresentation of players born in the younger year, chi-square analyses were conducted. Whether players belonged to the ‘younger’ or ‘older’ age cohort was determined as follows: the FIFA Under-17 World Cup takes place every second year which is always an odd year. This has an important implication for participating players namely that players born in odd years belong to the younger 1-year age group for a considerable amount of time and, consequentially, players born in even years belong to the oldest age group in the respective tournament. To test for differences among the observed and expected distribution of players’ birth years, the whole tournament sample (1971–1996; *n* = 736) was categorized into either older or younger age cohorts, according to birth year and, importantly, according to players constituent year timing at the point in time they potentially participated in the Under-17 World Cup. To explore the differences between even (older constituent year) and odd (younger constituent year) birth years (*x*-axis) in relation to the number of World Cup players born in the respective years (*y*-axis), we used a graphical exploratory approach. We wanted to investigate whether any of the age cohorts, either the older or the younger 1-year age group in the multi-year age band, would show a higher number of participating players. Therefore, participants were grouped into 2-year age bands based on the timing of when they would have been eligible to participate in the Under-17 World Cup. The oldest participant was born in 1976 while the youngest one was born in 1996. As such, the oldest represented former 2-year age band is 1976/77 and the youngest 2-year age band is 1996/97. However, we did not isolate only those players from our sample who actually participated in a junior World Cup.

To determine whether effects vary by confederation, national team players were distributed into continental groups based on the continental grouping during the preliminary rounds. Consequently, the sample was subdivided in five groups: UEFA (representing Europe, 13 teams), CAF (Africa, 5 teams), AFC (Asia, 4 teams incl. Australia), CONMEBOL (South America, 6 teams), CONCACAF (North and Central America and the Caribbean, 4 teams). No team from the Oceania Football Confederation was represented at the World Cup in Brazil. Additionally, the *cultural continents* approach (Kolb, 1962) differentiating between North and Sub-Saharan Africa presented in Study 1 was utilized. Examinations of carry-over and relative age effects were based on the assumption that birthdates were distributed equally across the months of a year (Helsen et al., 2005). Since the number of players being 2 or 3 years under age at the time of their first Under-17 World Cup participation was shown to be very small in Study 1, we did not include this possibility in the analysis.

### Statistical Analyses

IBM SPSS Statistics v23.0 and G^∗^Power 3.1 were used for the statistical analyses. To test for an interaction of constituent year and within-1-year effects, a MFA was conducted. Differences between the observed and expected distributions among the relative age quartiles and birth years were considered using chi-square analyses, with the effect size *w* reported (Sullivan and Feinn, 2012). Again, an equal distribution of births across all months and years was assumed.

### Results Study 2

The current study investigated the pervasiveness of within-year effects in adult elite soccer and potential cross-over effects from players’ constituent year timing when they originally participated in the Under-17 World Cup and therefore from talent development systems.

#### Global Results

To test for an interaction of players’ constituent year and within-year effects, a MFA was conducted. The MFA revealed no significant interaction among the overall 2014 World Cup sample, χ^2^ (3, *n* = 736) = 3.22, *p* = 0.36. In a second step, we ran a chi-square analysis to replicate previous findings on within-year effects in elite adult soccer and to test for potential long-term effects of age grouping structures existing during earlier stages of talent development. A significant overall within-year effect was revealed among the World Cup participants, χ^2^ (3, *n* = 736) = 11.34, *p* = 0.01, *w* = 0.14. An overrepresentation was found for quartile 1 with 29.8%, while quartile 2 (24.7%), quartile 3 (24.5%), and quartile 4 (21.1%) were under-represented.

**FIGURE 2:**
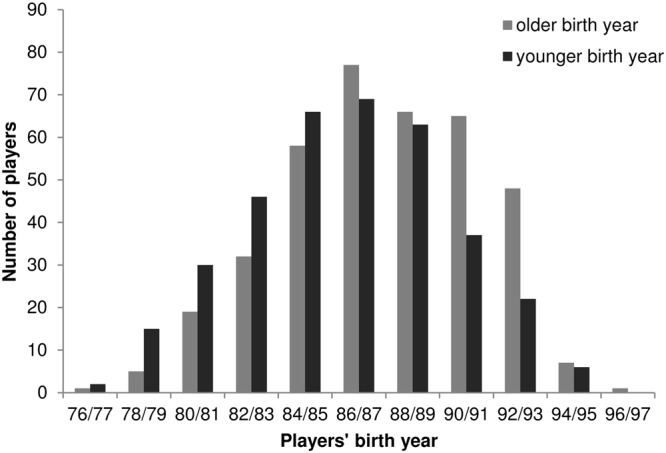
**Distribution of birth years among players participating in the 2014 World Cup**.

Testing for carry-over effects regarding overrepresentations of players born in the year that constituted the older 1-year age band at the time players were eligible to participate in the Under-17 World Cup, chi-square analyses revealed no significant results for the overall World Cup sample of soccer players born between 1976 and 1996, χ^2^ (1, *n* = 736) = 0.658, *p* = 0.42. However, **Figure 2** reveals that players are not distributed equally across the represented birth years. The distribution in form of an inverted U-curve has its maximum at birth years 1986/87 representing the largest number of players. Furthermore, a change in the number of players representing the older and the younger birth years at the time of players’ potential Under-17 World Cup participation was located at this point: for players born between 1976 and 1985 a significant overrepresentation of players born in the odd year and therefore members of the former younger 1-year age cohort was found, χ^2^ (1, *n* = 275) = 7.36, *p* = 0.007, *w* = 0.16. This trend changed among players born 1986 and after. Among players born between 1986 and 1996, a significant overrepresentation of players born in the older birth year of the 2-year age band was found, χ^2^ (1, *n* = 461) = 9.74, *p* = 0.002, *w* = 0.15. Age bands characterized by an on-time constituent year timing at a major event in the FIFA talent development system outperformed the younger age bands in number of players nominated for the tournament in the youngest six 2-year samples (birth years 1986/87 until 1996/97).

#### Differentiating between Confederations

To analyze the overall within-year effect in more detail, the results were differentiated between continental confederations. The MFA did not reveal any significant interactions among the sample from each confederation or for the two African cultural continents. However, an overall significant difference regarding players’ birth quartile distribution was observed between continents with a chi-square test on this cross table. A significant within-year effect was found for the UEFA, χ^2^ (3, *n* = 299) = 12.51, *p* = 0.01, *w* = 0.20, and the teams of the AFC, χ^2^ (3, *n* = 92) = 13.65, *p* < 0.01, *w* = 0.38, where an astonishingly low amount (i.e., less than 10%) was born in quartile 4. For the continental soccer confederations of South America (CONMEBOL) and North-Central America (CONCACAF), results showed a trend toward an expected relative age distribution, but results were not significant, South America, χ^2^ (3, *n* = 115) = 4.03, *p* = 0.26, North and Central America, χ^2^ (3, *n* = 92) = 0.96, *p* = 0.81. Interestingly, the descriptive trends differed considerably between South America, North-Central America, and Africa (CAF): while the American confederations showed an expected relative age distribution, Africa showed an overrepresentation in quartile 4, χ^2^ (3, *n* = 115) = 5.17, *p* = 0.16, *w* = 0.21. The results for the CAF did not reach significance, but 34% of African players were born in quartile 4, which is the highest value per quartile. Differentiating between North African nations and the sub-Saharan nations, results showed contrary trends: the North African sample (represented by Algeria only) showed an overrepresentation of quartile 1 born players [Q1: 43.48% (*n* = 10); Q2: 21.74% (*n* = 5); Q3: 8.7% (*n* = 2); Q4: 26.08% (*n* = 6)] and the sub-Sahara sample showed an inversed birthdate distribution with quartile 4 being overrepresented [Q1: 18.47 (*n* = 17); Q2: 22.83 (*n* = 21); Q3: 22.83 (*n* = 21); Q4: 35.87 (*n* = 34)], but results did not differ significantly from an even distribution.

### Discussion Study 2

Even though within-year effects have been shown to decrease with increasing age in many sports (Cobley et al., 2009) these effects have frequently been shown to exist among adults in high-level soccer (e.g., Richardson and Stratton, 1999; Helsen et al., 2012). Referring back to the original purpose of our study, previous findings on relative age effects in soccer were replicated in terms of a general within-year effect existing among the overall sample of players participating in the World Cup in 2014. Given the fact that within-year effects were demonstrated for the Under-17 youth developmental teams (Williams, 2010) representing the recent adult World Cup participants’ age groups and that these effects have also been demonstrated in the senior system in the current study, carry-over effects might exist. Although high-level performance at a young age is no reliable predictor for a nomination to adult national soccer teams (Williams and Reilly, 2000; Ostojic et al., 2014), young players’ skewed birthdate distributions seem to be a traceable attribute describing the respective pool of players selected for national teams.

Another aim of Study 2 was to test whether birthdate distributions differed between confederations. Interestingly, results demonstrated considerable variations between confederations and geographical regions with regard to the effects’ strength and direction. These results reinforce the emerging evidence that relative age is still an important constraint on the development of expertise in soccer, especially within the UEFA and the AFC where significant effects were revealed. Typical relative age distributions were shown for the American confederations as well as for North African participants, but results were not significant (eventually partly due to too small sample sizes). Findings on inverse birthdate distributions for the CAF and sub-Saharan Africa were replicated (Williams, 2010; Sallaoui et al., 2014) since trends toward an overrepresentation of players born late in the competition year were shown. However, results did not reach significance. Results indicate that overrepresentations of relatively older players still exist at the highest level of adult soccer competition but vary by confederation.

The current study also explored the interplay between players’ former constituent year timing, determined by the time they were eligible to participate in the Under-17 World Cup, and the number of players from each 1-year age cohort represented in the World Cup 2014. The aim of the study was to test for a potential crossover of constituent year effects shown in junior national teams (e.g., the Under-17 teams explored in Study 1). Testing for carry-over effects regarding overrepresentations of players born in the year that constituted the older 1-year age band at the time players were eligible to participate in the Under-17 World Cup, analyses revealed no significant results for the overall sample of soccer players born between 1976 and 1996. The distribution of birth years takes the form of an inverted U-curve with a maximum at the years 1986/87 (see **Figure 2**). This finding is in line with previous findings on soccer players’ performance development indicating that peak performance is generally reached in soccer at the age of 27–30 years (Boone et al., 2012). The decline of the number of players born before 1986 might be an early trend toward the results revealed by Medic et al. (2013), namely that being characterized by a relatively younger constituent year might become more beneficial with increasing age. For players born between 1986 and 1996 a significant overrepresentation of players with ‘on time’ constituent year timing was found. These results indicate that it takes players who were categorized to a younger age group by the system years to overcome the negative consequences that affected them. Taking a closer look at the distribution of birth years, another striking detail comes to light that contradicts the assumption that this distribution is just due to a linear increase of performance with increasing age up to a certain age: the number of players born between 1990 and 1992 does not decline as expected. While 65 players were born in 1990, only 37 players were born in 1991. However, instead of declining further the next year (i.e., with younger age), the number of players born in 1992 increases again suggesting that these players still benefit from being among the oldest age group during the years when important talent development championships took place. The Under-17 World Cup is undoubtedly a major event in the career development of youth soccer players. Not being ‘on time’ with regard to the constituent year timing in the year the junior World Cup takes place, the younger age group might have received less attention, support, and development opportunities compared to their teammates born 1 year earlier – although it is worth noting that many nations provide additional development teams such as Under-16 teams (Helsen et al., 2005). Importantly, these considerations remain largely hypothetical and require further investigation. It is also important to note that additional selection procedures and crucial stages of talent development are located between a young player’s nomination for the Under-17 World Cup and a potential participation among the adult World Cup such as the Under-20 World Cup and different continental championships such as the European Under-19 championships or the Africa Under-20 Cup of Nations.

In contrast, no long-term consequences or carry-over effects were revealed for the interaction between different relative age effects shown among elite junior soccer players in Study 1. A possible reason is that the interaction might wash out over the course of the years. It is possible that the small number of players (15%) born in the younger constituent year were early maturers, which may affect their career development: research suggests early maturing boys tend to dropout and that late maturing boys are favored as the level of performance increases (Ostojic et al., 2014). As such, it would be interesting to follow younger constituent year players’ progression through the development system. Furthermore, the investigated samples might include too many undetected constraints (Wattie et al., 2015) affecting the role of relative age on lower organization levels located in the respective national talent development and sport systems. At the present moment no explanation can be provided for the interesting birth year distribution among adult World Cup participants. Players’ progress to peak performance which is usually reached by the age of 27–30 years in soccer (Boone et al., 2012) is a plausible approach to explain the distribution curve showing the largest cohorts of participating players were aged 26–29 years. However, this approach might not entirely explain the irregular changes in the birth year distribution and the enduring trends toward a typical within-year effect birthdate distribution in the World Cup sample.

## General Discussion

Initially reported in sport in the 1980s, there have been few attempts to examine whether talent selection strategies have changed over time in light of the considerable amount of studies investigating relative age effects, especially in soccer. Helsen et al. (2012) compared birthdate distributions of professional soccer players in 10 European countries over a 10-year period (2000/01 and 2010/11). Results did not indicate any change in within-year effects in professional soccer during the time period investigated. In line with findings from the current study, these outcomes emphasize the robust nature of this phenomenon and highlight the influence of relative age effects on current talent selection procedures, which seem profoundly biased toward a young player’s physical attributes (Helsen et al., 1998a).

For future investigations on the different types of relative age effects (Schorer et al., 2013), a more complete overview of cut-off dates would be useful. One problem in our analyses in both studies was that while January 1st is widely used as a cut-off date, there are probably some exceptions. Although FIFA has defined January 1st as the cut-off date in the junior tournament regulations^4^ no study has evaluated whether this cut-off date is realized consistently at earlier or more regional stages of talent development in soccer. For example, we considered different ways to receive information on current cut-off dates used in Africa but gathering this information was more difficult than anticipated. Due to this lack of information, the results for the CAF should be interpreted carefully and require further clarification.

The overrepresentation of players born in quartile 4 in sub-Saharan Africa might also be influenced by potential errors in reporting actual birth dates (Williams, 2010). There is some evidence (Dvorak et al., 2007) challenging whether the obtained birthdates are accurate (for possible mechanisms that might lead to errors in reporting an individual’s birth date see Ndong et al., 1994; Akande and Sekoni, 2005). Nevertheless, it might be worth considering alternative interacting factors to explain the emergence of conspicuous birthdate distributions. If the overrepresentation of quartile 4 in Africa is based on different strategies with regards to talent selection and development, these strategies would be important to explore.

While the internationality of this sample is a captivating characteristic, it also constitutes the main challenge and the major limitation to these studies. Due to the general preponderance of studies investigating the role of relative age in Europe and North-America (Cobley et al., 2009) little is known about the system-specific particularities that could influence different types of relative age effects. To reveal the mechanisms underlying the varying patterns of birthdate distributions replicated in the current studies, future research should more deeply investigate and compare selected systems to close existing research gaps on the role of relative age in soccer. As requested by Wattie et al. (2015), future studies could investigate the multiple constraints within different talent development systems in soccer. For example, studies might shed light on the importance of physical attributes to a national team’s philosophy, players’ learning environment and amount of hours of practice and deliberate play (Côté et al., 2003), and coaches’ as well as talent scouts’ awareness of the possible impact of relative age (Helsen et al., 2005). A comparison of findings from different talent development systems may provide insight into the possible impact of the factors mentioned above on different birthdate distribution patterns in soccer (Williams, 2010; Wattie et al., 2015).

In summary, the pervasiveness of within-year effects on the highest levels of sport (i.e., soccer competition) and the need to understand the underlying mechanisms have been demonstrated once again. The investigation of the Under-17 FIFA World Cup participants from 2007 until 2015 in Study 1 revealed advantages for relatively older players over the past five competitions. The birthdate distributions of the teams participating in these championships showed a clear bias in favor of players born in the early months of the selection year. A reverse within-year effect was repeatedly found for the African confederation. The reasons for the emergence of these findings are not yet fully understood. Although non-accurately reported birthdates might be an explanation, future research should also explore alternative explanations. Nevertheless, a geographic differentiation of birthdate analyses emerged again as a useful means to uncover the nuances of different relative age effects. Additionally, system structures seem to foster constituent year effects in Under-17 tournaments.

The persistence of within-year effects was replicated in Study 2. Furthermore, this investigation provides some evidence that players’ constituent year timing at earlier stages of talent development has a long-term impact on players’ career development and participation rates. Future studies might consider Under-20 World Cups as a further point in time in young players’ career development to eventually address the role of players’ constituent year timing relative to the timing of important tournaments. Taken together, findings from these studies underline the increasingly complex phenomena of relative age effects in sport. We hope the pervasiveness and complexity of this phenomenon will motivate researchers and practitioners to search for strategies and solutions for reducing this inequality. As such, research in this area should try to find a way to provide more equal opportunities for all sport participants regardless of birth date (Baker et al., 2010).

## Author Contributions

CS, NW, JB, WH, and JS substantially contributed to the study design of the work, to data analysis as well as to the interpretation and discussion of the results in this work; CS, NW, JB, WH, and JS drafted the manuscript and revised it critically. All authors CS, NW, JB, WH, and JS gave their final approval of the current manuscript version to be published and agree to be accountable for all aspects of the work in ensuring that questions related to the accuracy or integrity of any part of the work are appropriately investigated and resolved.

## Conflict of Interest Statement

The authors declare that the research was conducted in the absence of any commercial or financial relationships that could be construed as a potential conflict of interest.

## Footnotes

1 https://de.fifa.com/u17worldcup/

2 https://www.fifa.com

3 http://www.fifa.com/worldcup/organisation/index.html

4 www.fifa.com
